# Green Tea Catechins for Prostate Cancer Prevention: Present Achievements and Future Challenges

**DOI:** 10.3390/antiox6020026

**Published:** 2017-04-05

**Authors:** Valeria Naponelli, Ileana Ramazzina, Chiara Lenzi, Saverio Bettuzzi, Federica Rizzi

**Affiliations:** 1 Department of Medicine and Surgery, University of Parma, Via Gramsci 14, Parma 43126, Italy; valeria.naponelli@unipr.it (V.N.); ileana.ramazzina@unipr.it (I.R.); chiara.lenzi1@studenti.unipr.it (C.L.); 2 Centre for Molecular and Translational Oncology (COMT), University of Parma, Parco Area delle Scienze 11/a, Parma 43124, Italy; 3 National Institute of Biostructure and Biosystems (INBB), Viale Medaglie d’Oro 305, Rome 00136, Italy; 4 Fondazione Umberto Veronesi, Piazza Velasca 5, Milan 20122, Italy

**Keywords:** green tea catechins, prostate cancer, chemoprevention, nanochemoprevention, antioxidant, mechanism of action

## Abstract

Green tea catechins (GTCs) are a family of chemically related compounds usually classified as antioxidant molecules. Epidemiological evidences, supported by interventional studies, highlighted a more than promising role for GTCs in human prostate cancer (PCa) chemoprevention. In the last decades, many efforts have been made to gain new insights into the mechanism of action of GTCs. Now it is clear that GTCs’ anticancer action can no longer be simplistically limited to their direct antioxidant/pro-oxidant properties. Recent contributions to the advancement of knowledge in this field have shown that GTCs specifically interact with cellular targets, including cell surface receptors, lipid rafts, and endoplasmic reticulum, modulate gene expression through direct effect on transcription factors or indirect epigenetic mechanisms, and interfere with intracellular proteostasis at various levels. Many of the effects observed in vitro are dose and cell context dependent and take place at concentrations that cannot be achieved in vivo. Poor intestinal absorption together with an extensive systemic and enteric metabolism influence GTCs’ bioavailability through still poorly understood mechanisms. Recent efforts to develop delivery systems that increase GTCs’ overall bioavailability, by means of biopolymeric nanoparticles, represent the main way to translate preclinical results in a real clinical scenario for PCa chemoprevention.

## 1. Introduction

Prostate cancer (PCa) is the second most common malignancy and a major cause of cancer deaths in men [1,2]. The increasing trend in PCa incidence reported over the last two decades, as well as differences in the geographic distribution of this value among different countries, show a good parallelism with differences in the use of the Prostate Specific Antigen (PSA) assay for PCa diagnosis. The highest number of screenings occurs in Western countries, where the highest incidence is found [3]. In contrast, the incidence of PCa is much lower in Asia and Africa, where PSA screening has not been widely adopted until now. Apart from differences caused by a scarce diffusion of the PSA test in some countries, which may lead to a reduced detection of latent PCa, different dietary habits and lifestyles may independently contribute to the highest risk of developing PCa in elder men who live in Western countries. In support of this concept, we have to keep in mind that there was already more than a 50-fold difference in international PCa incidence rates across Eastern and Western countries in 1980, before the PSA test was introduced [4]. To further emphasize the role of lifestyle in PCa development, migratory studies have found that Asian men who relocate to the United States and adopt a western lifestyle have a significantly higher risk to develop PCa than their native Asian counterparts [5,6]. PCa is an age related disease, highly heterogeneous and largely incurable at metastatic stages. The vast majority of prostate adenocarcinomas are characterized by a long latency period, which is generally clinically silent. Accordingly, only a few patients that receive a diagnosis of PCa will experience invasive and eventually metastatic cancer and will die of the cancer. The prognosis of high grade PCa is often unfavorable, and the surgical management of the localized disease is related to adverse events that negatively impact on the patients’ quality of life. For these reasons prevention may be the best approach to reduce PCa morbidity and mortality at the present time [7].

Among many the dietary agents investigated for chemopreventive properties against PCa, green tea derived polyphenols (GTPs) have received much attention [8]. In many European countries, the consumption of tea is occasional, whereas in Asian countries tea is extensively consumed as a beverage and has been associated with a reduced risk of advanced PCa [9].

**Figure 1:**
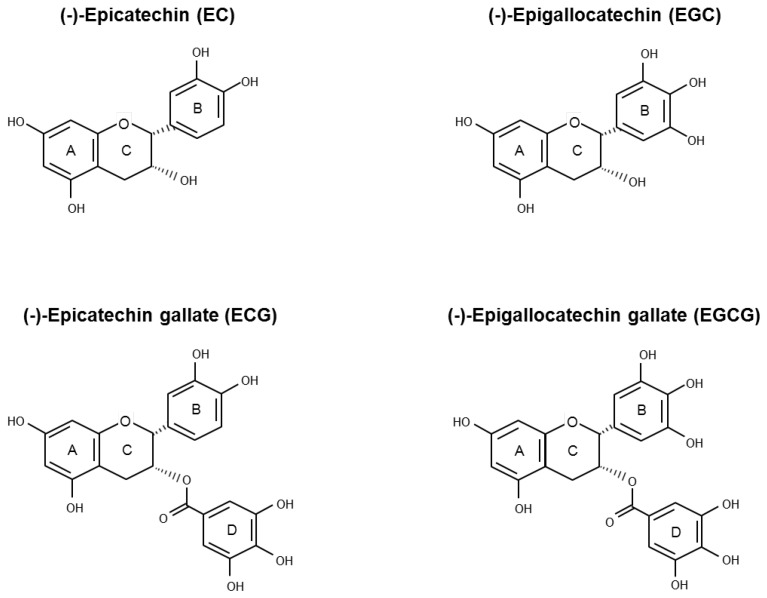
Chemical structure of the four main green tea catechins (GTCs): (−)-epicatechin (EC), (−)-epigallocatechin (EGC), (−)-epicatechin gallate (ECG), and (−)-epigallocatechin gallate (EGCG).

Green tea is sourced from *Camellia sinensis* (L.) O. Kuntze (*Theaceae*), the same plant from which black and oolong teas are derived. The various tea types are classified on the basis of their manufacturing processes that influence taste, colour, and polyphenolic content composition. During green tea production, the endogenous oxidase enzymes in tealeaves are heating inactivated so that GTPs are preserved [10]. Flavonoids are the major active polyphenolic components of dried green tea extracts, in which flavan-3-ols account for more than 10% by weight [11]. Healthy properties of green tea have been associated with a family of flavan-3-ols called catechins. In decreasing order of abundance, the main green tea catechins (GTCs) are epigallocatechin gallate (EGCG), epigallocatechin (EGC), epicatechin gallate (ECG), epicatechin (EC), gallocatechin (GC), and catechin (C) (Figure 1).

EGCG accounts for about 40% of the total catechin content in green tea leaves and is the focus of much of the research on the health beneficial properties of green tea and green tea derived preparations. EGCG has significant growth inhibitory properties in PCa cells, with an observed half maximal inhibitory concentration (IC_50_) ranging from 40 μM to 80 μM, depending on the cell line used, the length of treatment, and the administration protocol [8].

It is noteworthy that the cytostatic action of GTCs is cancer specific, since EGCG is capable of inducing growth arrest both in SV-40 immortalized prostate epithelial cells and in PCa cells at concentrations that do not cause any toxic effect in normal human prostate epithelial cells [12,13,14].

The inhibition of PCa by GTCs and the prevention of tumor progression to the metastatic stage have been consistently documented in animal models that spontaneously develop PCa by many independent research groups [12,15,16]. Similar results have been documented also in men bearing precancerous lesions and achieving standardized doses of GTCs for one year [17,18]. The main obstacle to fully translate the more than promising results observed in preclinical models to large clinical trials is the identification of a reliable mechanism of action in vivo. Due to the very low bioavailability of dietary GTPs and standardized GTCs preparation, the mean plasmatic concentration reached by EGCG is between 50 to 100 times lower than the concentration used in vitro [19].

Recent works focus their attention on molecular or cellular mechanisms that are specifically altered during the process of cell transformation and that might be the in vivo target of GTCs, even at nanomolar concentrations.

**Figure 2:**
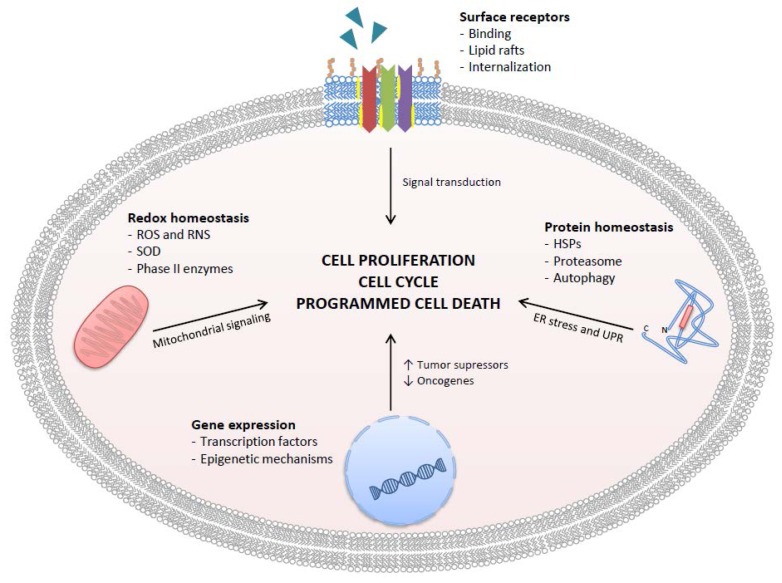
Targets of GTCs in human PCa cells. GTCs inhibit cancer cell proliferation, cause cell cycle arrest, and promote programmed cell death by four main mechanisms: inhibition of ligand-receptor complex formation, redox homeostasis alteration, gene expression control, and protein homeostasis alteration. ROS = reactive oxygen species; RNS = reactive nitrogen species; SOD = superoxide dismutase; HSPs = heat shock proteins.

This review will start discussing the antioxidant/pro-oxidant properties of GTCs, then will discuss recent experimental achievements on specific mechanisms of action that involve direct interaction with surface receptors and relevant intracellular targets, and then will move to describe observational and interventional studies that investigated the effectiveness and safety of GTCs in a real clinical setting (Figure 2). Attention will be paid to GTCs metabolism and bioavailability and, finally, to recent advances in the development of nanodelivery systems that improve GTCs bioactivity through increased stability, improved absorption, and reduced excretion.

## 2. Effects of GTCs on Cellular Redox Homeostasis and Antioxidant Signaling

In both physiological and pathological conditions, reactive oxygen and nitrogen species (ROS and RNS, respectively) are produced. These reactive species are essential for cell survival and are involved in normal processes such as adaptation to hypoxia, regulation of autophagy, immunity, differentiation, and longevity [20]. A moderate ROS increase triggers the hormesis effect, an adaptive paradigmatic response to reactive species that helps cells to cope with oxidative stress through the activation of specific redox-cellular signaling pathways [21,22,23]. When the production of ROS and NOS overwhelms the cell capacity to scavenge them, oxidative stress occurs, leading to damage to biological molecules, loss of function, and eventually cell death.

The redox homeostasis imbalance is considered to be an important mechanism in promoting different diseases, including cancer onset and progression. Chronic exposure to elevated ROS concentration has been associated with PCa development [24]. Different studies have revealed that oxidative stress markers are higher in PCa cells (both in cell lines and human samples) in comparison to their non-pathological counterparts [24,25]. The cellular redox imbalance cooperates with other factors associated with PCa etiogenesis such as aging, androgen receptor activity, hormonal imbalance, and chronic inflammation. Different detoxification enzymes and antioxidant molecules are involved in maintaining the redox balance inside the cell. One of the most important mechanisms involved in superoxide detoxification involves the enzymes superoxide dismutases (SODs) that produce H_2_O_2_, which is in turn switched-off by catalase or glutathione peroxidase activity. Along with enzymatic activities, different endogenous compounds like glutathione, plasma protein thiols, and iron-binding proteins are needed to maintain the oxidative balance. Dietary components, like vitamins and polyphenols also contribute to maintaining intracellular redox homeostasis [26,27]. An increasing number of studies have demonstrated that GTPs are strong antioxidant compounds [21,28]; nonetheless GTPs are also able to exert pro-oxidant effects. The antioxidant/pro-oxidant behavior of GTPs greatly depends on many experimental conditions, such as the final concentration of use, temperature, pH, presence of metal ions, and differences in culture media composition [21,29,30,31]. All these variables make it very difficult to compare results, which are sometimes contradictory, published by various authors so far.

### 2.1. Effects of GTCs on Intracellular ROS Production

GTCs backbone (Figure 1) is characterized by the presence of two benzene rings (A- and B-ring) with two or more hydroxyl groups attached and a dihydropyran heterocycle (C-ring). The conjugation of hydroxyl groups on the C-ring with gallic acid (D-ring) leads to the formation of EGCG and ECG. The antioxidant properties of the GTCs are due to the presence of both the phenolic groups and the galloyl moiety, which allow electron delocalization and free radical quenching ability. According to their structural features, EGCG and ECG are, among the four most abundant GTCs, the strongest antioxidants [27,32]. However, GTCs may undergo auto-oxidation reactions, leading to the formation of catechin dimers and free radicals that may be responsible for the pro-oxidant action observed in vitro after polyphenol administration. The level of generated H_2_O_2_ is positively correlated with exposure time and the polyphenol concentration used. Incubation of 100 μM EGCG or EGC in different cell culture media for 1 h determined the generation of H_2_O_2_, ranging between 50.7 ÷ 71.6 µM and 98.1 ÷ 115 µM, respectively. However, at the concentration of 10 μM (a value that is about 10-fold higher than the values achievable in human plasma after ingestion of 800 mg of GTCs), the amount of generated H_2_O_2_ is very low [33]. Of note, the administration of EGCG up to 50 µM in different cell culture media in the presence of CHO cells exerts mild effects on cell viability, indicating that living cells possess efficient defense systems against H_2_O_2_ produced by GTCs in aqueous solutions [34]. Transition metals are able to initiate the catechins’ auto-oxidation reaction, also promoting the formation of catechins-metal ion complexes. Depending on the experimental model, these mechanisms have been correlated both to an antioxidant or a pro-oxidant behavior of GTCs [21,28]. Zinc is required for normal prostate metabolism due to its ability to inhibit aconitase activity, determining the excretion of citrate into seminal fluid. The ability of Zn^2+^ to accumulate in the prostate gland is lost during PCa progression [35]. Experimental data have shown that, in PCa cell lines, a mixture of Zn^2+^ and EGCG enhances both the growth inhibitory effect and the free radical scavenging ability with respect to EGCG alone. Moreover, Zn^2+^ enhances EGCG effects in terms of reduction of mitochondrial membrane potential and caspase-9 activation [36,37,38]. In PC-3 ML and in IBC-10a primary prostate cell lines, the administration of EGCG (up to 60 µM) determines a reduction of mitochondrial membrane potential and integrity, followed by apoptosis without increase in ROS production [39]. Chung and colleagues have observed that the incubation of DU145 cells with green tea extract (50 μg/mL) or pure green tea compounds (100 μM) for 48 h induces apoptosis through a peroxide and superoxide anions level increase and mitochondrial depolarization [40]. Kanwal and colleagues treated LNCaP cells with EGCG (20 µM) or Polyphenon E^®^ (10 µg/mL), a standardized GTCs-enriched mixture that is caffeine free, containing 80% to 98% total catechins by weight. The authors have demonstrated that the 72 h-treatments make cells more resistant to both H_2_O_2_-induced oxidative stress and DNA damage [41].

We studied the effects of increasing doses of Polyphenon E^®^ on ROS production and cell viability in PCa cell lines. We reported that concentrations of Polyphenon E^®^ yielding a significant increase in ROS production (5 ÷ 20 µg/mL and 5 ÷ 50 µg/mL for PNT1a and PC3, respectively) displayed marginal effects on cell proliferation. A similar effect on ROS production was obtained by treating cells with H_2_O_2_ (10 µM and 50 µM for PNT1a and PC3, respectively). Higher concentrations of Polyphenon E^®^ that caused a significant reduction of cell proliferation (25 ÷ 35 µg/mL and 75 ÷ 145 µg/mL for PNT1a and PC3, respectively) produced an amount of ROS below that of untreated cells or cells given H_2_O_2_. There is not good correlation between ROS induction and cell death in PCa cells following Polyphenon E^®^ treatment. Therefore, it is very unlikely ROS production plays a direct role in GTCs induced cell death. Nonetheless, this process is strictly associated with the induction of endoplasmic reticulum stress, a mechanism that impacts on protein homeostasis, finally leading to cell death [42].

### 2.2. Effects of GTCs on SOD Activity

Apart from the direct effect on ROS production, EGCG is also involved in the modulation of SODs activity. Many studies, reviewed in [43], suggest that manganese superoxide dismutase (MnSOD) participates in the maintenance of mitochondrial integrity in cells exposed to oxidative stress. Morrissey and colleagues have shown that EGCG treatment (20 µM for 72 h) of NRP-154, a tumorigenic rat prostate epithelial cell line, determines a reduction of the expression of MnSOD, but not of catalase, concomitantly with apoptosis induction [43]. On the contrary, in DU145 cells, MnSOD expression was not affected by the administration of 3 µM EGCG, while a significant reduction was observed after treatment with ionizing radiation (IR). In the same experimental model, the combination of EGCG and IR significantly reduced IR-induced apoptosis, together with an increase of MnSOD expression. Therefore EGCG might promote radiotherapy resistance, inducing MnSOD [44]. In contrast, in Lobund-Wistar rats, the chronic consumption of green tea decreases the tumor incidence of genitourinary tissues in association with an increase of MnSOD expression, without effects on DNA damage, lipid peroxidation, and lipofuscin deposition [45]. Treatment of TRAMP (Transgenic Adenocarcinoma of the Mouse Prostate) mice for 13 weeks with EGCG (administered by gavage at a dosage of 200 mg/kg body weight), starting at 7 weeks of age, was associated with a significant reduction of severity/focalness of the histopathological lesions observed in the ventral lobe of the murine prostate. These effects are unlikely due to a direct antioxidant effect of EGCG; indeed the authors observed only a slight reduction in plasma H_2_O_2_ concentration and no significant effect on SOD expression [46]. Later on, in the same experimental model, the authors found that EGCG reduced the DNA oxidative damage marker but had no effects on the protein and lipid peroxidation markers [47].

### 2.3. Effects of GTCs on -RNS Production

Nitric oxide is a free radical produced by three different isoforms of nitric oxide synthase (NOS). NO**^•^** actions are mediated through cGMP-dependent or cGMP-independent pathways. Moreover, NO^•^ at low concentrations can interact with different cellular compounds like other free radicals, proteins, and DNA. NO**^•^** exerts a dual role in cancer cells, which is dose dependent. At low concentrations (less than 100 nM), NO**^•^** is associated with tumor cell proliferation and angiogenesis; at high concentrations (more than 500 nM), NO**^•^** exerts a cytotoxic effect [48]. The anticancer effect of EGCG, through the inhibition of NO**^•^** production, has been observed in many different tumors [48,49,50,51]. In TRAMP mice who received 0.06% EGCG in tap water ad libitum for seven weeks starting at weaning, a reduction of the High Grade Prostate Intraepithelial Neoplasia (HG-PIN) incidence was observed concomitantly with a significant down-regulation of iNOS [52].

## 3. Specific Molecular Mechanisms of Action of GTCs

Apart from their antioxidant/pro-oxidant activity, GTCs have a high affinity with many biomolecules, including phospholipid bilayers, proteins, and nucleic acids. It has been shown that GTCs interact with cell surface receptors or enter the cells and directly interact with specific intracellular molecular targets [53,54]. Once GTCs have interfered with the ligand-receptor binding, or more generally with receptor activation, the effects are reflected in intracellular signaling pathways that control cell proliferation/cell death through the activation/inhibition of regulatory proteins (kinases/phosphatases) or effector molecules. GTCs interfere with gene transcription both by direct regulation of transcription factors and by epigenetic mechanisms that produce modifications of chromatin accessibility. GTCs may regulate gene expression also at the post transcriptional level by a mechanism that involves the expression of particular miRNAs (microRNAs). Of particular note, in the prostate, an endocrine tissue characterized by huge protein synthesis activity, GTCs have been shown to interfere with molecules, cellular compartments, or mechanisms reputed to control protein homeostasis, such as molecular chaperons, the endoplasmic reticulum (ER), and the main protein degradation mechanisms.

### 3.1. Cell Surface Receptors

GTCs interfere directly or indirectly with the formation of the ligand-receptor complex of several cell surface receptors [55,56]. Three different mechanisms have been proposed to explain the effect of GTCs on the regulation of the activity of cell surface receptors and their signaling pathways. The first mechanism proposes that GTCs physically prevent the interaction of the ligand with its cognate receptor by completely covering the cell surface through a “sealing effect” [57] or binding to the ligand [58,59] or competing with the ligand for the receptor [60].

The second mechanism involves the suppression of the intrinsic activity of lipid rafts-associated signaling proteins by lipid rafts disruption [55,61,62]. The third one prevents the interaction between receptor and ligand, promoting receptor clearance through physical sequestration inside endosomal vesicles [63].

Receptor tyrosine kinases (RTKs) have been recently identified as targets of GTCs in cancer cell growth inhibition [55,64,65]. The binding of a growth factor or a cytokine to the extracellular domain of RTKs induces the dimerization and autophosphorylation of the RTKs on specific tyrosine residues and the activation of downstream intracellular signaling that includes Ras/ERK/MAPK and PI3K/Akt. Ras/ERK/MAPK and PI3K/Akt activation triggers a cascade of molecular events involving enzymes, proteins, and transcription factors that have been extensively reviewed in [66]. The activation of cell surface RTKs controls many key important processes in normal cells such as cell proliferation, differentiation, survival, and migration. These receptor-associated pathways have been found altered in tumor cells, including PCa [55,66,67,68,69]. The RTKs family is comprised of epidermal growth factor receptors (EGFRs), fibroblast growth factor receptors (FGFRs), insulin-like growth factor receptors (IGF-R), platelet-derived growth factor receptors (PDGFRs), vascular endothelial growth factor receptors (VEGFRs), and hepatocyte growth factor receptors (HGFRs), also known as Met. The most effective inhibition toward RTKs is exerted by EGCG, followed by ECG and EGC [67].

GTCs administration inhibited EGFR activation in several cancer cell lines, even at concentrations as low as 1 µg/mL [70,71,72,73]. Liang et al. demonstrated that EGCG directly prevents the interaction between ligand and EGFR necessary for the tyrosine kinase activity in human epidermoid carcinoma cells (IC_50_ = 0.5 ÷ 1 µg/mL) [59].

Similar effects of GTCs have been observed towards insulin-like growth factor receptor 1 (IGF-R1) [74]. IGF-R1 is overexpressed in PCa [67]. Interestingly Li et al. demonstrated that EGCG competitively binds to the ATP binding site of IGF-R1, inhibiting its kinase activity in vitro (IC_50_ = 14 µmol/L) and in vivo [60]. The blockage of ligand-receptor binding has been suggested as the mechanism behind the inhibitory effect of GTCs on PDGFR and HGFR in multiple cell lines at concentrations of 1 ÷ 10 µM [58,75,76,77]. Tachibana et al. proposed that EGCG is a ligand of the 67-kDa laminin receptor (67-LR) with a dissociation constant (Kd) value of 0.004 µM [78]. 67-LR expression is elevated in cancer cells, where it acts as a cancer-specific cell death receptor. EGCG pro-apoptotic effects in multiple myeloma cells are abrogated by 67-LR silencing, suggesting that EGCG induced cell death is achieved through a 67-LR mediated mechanism [79]. Proof of the direct binding between EGCG and 67-LR has been recently provided by Shukla and colleagues. These authors have shown that the delivery of radioactive gold nanoparticles (NPs) functionalized with EGCG in PCa cells was mediated via the 67-LR. When the 67-LR was blocked by laminin or by anti-67LR antibody, the uptake of NPs was prevented [80]. Recent data have confirmed that EGCG undergoes oligomer formation by binding to the 67-LR [81]. In particular it has been identified a sensing motif on 67-LR, located within the peptide LR161-170, which is critical for the binding of EGCG [82].

The second mechanism proposed to explain the ubiquitous effect of EGCG on multiple receptors involves the disruption of lipid organization in the plasma membrane [61]. Lipid rafts are high ordered membrane structures, rich in cholesterol and sphingolipids, that regulate protein-protein interaction, receptor activation, and therefore, more generally, cellular signaling. EGCG alters the lipid organization of the plasma membrane and causes the rearrangement of lipid rafts [83], preventing the activation of cell surface receptors. In multiple myeloma cells, EGCG promoted apoptosis through lipid rafts clustering that, in turn, mediated the activation of 67-LR and triggered pro-death signaling [84,85,86,87]. GTCs inhibited RTKs activation through lipid rafts clustering. This mechanism has been reported for EGFR, HGFR, IGF-R1, and VEGFR [61,62,88,89,90,91].

Finally Adachi et al. found that the EGFR is quickly internalized inside endosomal vesicles in human colon cancer cells treated with EGCG (1 µg/mL for 30 min). The internalization makes the receptor inaccessible to epidermal growth factor and abrogates RTKs signaling cascade [63].

### 3.2. Effects of GTCs on Regulation of Gene Expression

Although the mechanisms of the cancer chemopreventive effect of GTCs have not been completely elucidated, there is evidence that EGCG administration results in the modulation of gene expression [12,92]. It has been demonstrated that EGCG modulates activity and expression of various transcription factors including Sp1, NF-kB, AP-1, STAT1, STAT3, and nuclear factor erythroid 2-related factor 2 (Nrf2) [27]. Among these, Nrf2 and NF-kB are two redox-sensitive transcription factors responding to inflammatory/oxidative stress stimuli that control the expression of many genes involved in PCa onset and progression. It is now accepted that free radicals act as redox cellular messengers, triggering signaling pathways able to influence the redox homeostasis. One of the most important cellular responses to cope with oxidative stress is the activation of Nrf2, which in turn activates a plethora of enzymes. Keap1, the Nrf2 inhibitor, is sensitive to redox imbalance. In presence of oxidants or electrophilic compounds, Keap1 dissociates from Nrf2, which then translocates to the nucleus and binds to electrophile-responsive element (EpRE) sequences, driving the transcription of phase II enzymes such as glutathione peroxidase (GPX), glutathione S-transferase (GST), NADPH quinone oxidoreductase 1 (NQO-1), and UDP-glucuronosyltransferase (UGT) [93,94]. Nrf2 also acts through Keap1 independent mechanisms [95,96]. It is important to highlight that GTCs are able to activate multiple pathways, such as MAPKs, PI3K, PKC, and NF-kB, which may play a crucial role in the Nrf2-mediated response to oxidative stress [93,95]. EGCG, and more generally GTCs, exert their anticancer effect in many different tumor models through the induction of a beneficial moderate oxidative stress, which in turn activates the Nrf2/EpRE response [93,97]. A direct target of Nrf2 is the gene GSTP1, which is downregulated in PCa. The effects of GTCs on GSTP1 re-expression are discussed in the next paragraph. NF-kB is constitutively activated in PCa cells, prostate premalignant lesions (intraepithelial tumors, PIN), and PCa tissues [98]. EGCG has been demonstrated to inhibit NF-kB transcriptional activity in DU145 and LNCaP cells, promoting apoptosis via Bax up-regulation and Bcl2 down-regulation [99,100]. Siddiqui et al. reported that the constitutive activation of NF-kB, observed during PCa progression, was prevented in TRAMP mice receiving 0,1% GTCs in drinking water from weaning, in comparison to water-fed age matched controls [101]. One of the targets of NF-kB is represented by matrix metalloproteinases (MMPs), a family of proteolytic enzymes that degrade collagene and other extracellular matrix proteins contributing to PCa progression. GTCs have been reported to inhibit various MMPs in PCa cells [99,102] and in TRAMP mice [16]. In silico molecular docking analyses have recently shown a strong direct interaction between EGCG and MMP-9, suggesting that EGCG might inhibit MMP-9 also by an NF-kB independent mechanism [103].

#### Epigenetic Mechanisms

Epigenetics generally refers to changes in gene expression and chromatin organization that are independent of alterations in the DNA sequence. Epigenetic phenomena are modifiable by environmental factors that include dietary habits [104]. Cancer development involves a complex multistep process caused by and associated with many genetic insults that include genetic mutations as well epigenetic alterations [105]. Many tumor suppressor and receptor genes have been reported to be hypermethylated and transcriptionally silenced during the development of various human cancers, including PCa [106]. The major epigenetic mechanisms that control gene expression are DNA methylation, histone modifications, and expression of noncoding regulatory micro RNA (miRNAs). Extensive in vitro experiments reviewed by Henning et al. in [104] have been performed in a variety of cancer cell lines to evaluate the effect of GTCs or, more generally, GTPs on DNA methylation. There is evidence that GTCs cancer preventive effect can be ascribed to the epigenetic reactivation of silenced gene through the inhibition of DNA methyltransferases (DNMTs) activity. Fang et al. demonstrated that EGCG binds to DNMT and competitively inhibits the enzymatic activity (Ki of 6.89 µM), yielding the reactivation of methylation-silenced genes in PC3 cells [107]. Molecular modeling and docking studies supported the binding of EGCG to DNMT3B in HeLa cells [108]. EGCG (5 ÷ 20 µM) or Polyphenon E^®^ (1 ÷ 10 µg/mL) treatments of different PCa cell lines have determined a dose- and time-dependent re-expression of GSTP1 enzyme concomitantly with the down-regulation of DNMT1 [41,106]. The re-expression of GSTP1, induced by treatment with EGCG or Polyphenon E^®^, may be, at least in part, responsible for free radical species quenching and the reduction of DNA damage associated with oxidative stress [41]. Two studies concluded that GTCs did not produce a significant effect on DNA methylation in PCa cell lines and in TRAMP mice [109,110]. Potential reasons for these discrepancies might be due to different methods of analysis used to evaluate DNA methylation, different cell lines used, or inefficacy of the treatment protocol. Nonetheless, Henning et al. fed SCID mice implanted with LAPC4 androgen-dependent PCa cells with brewed green tea (containing 0.075% GTPs) or water for 13 weeks. The authors found that tumor growth and the expression of DNMT1 were reduced in xenograft tumors excised from the green tea-fed mice in comparison to the controls [111]. These results support the concept that GTCs may produce epigenetic changes in vivo following long-term administration.

The direct inhibition of EGCG on DNMT1 is enhanced in the presence of catechol-O-methyltransferase (COMT). The mechanism proposed by Lee et al. to explain this additive effect is due to the alteration of the S-adenosyl-methionine (SAM) and S-adenosyl-homocysteine (SAH) intracellular ratio. Indeed, galloylated GTCs are methylated by COMT, which catalyzes the transfer of a methyl group from the donor molecule SAM that in turn forms SAH. By this mechanism, the SAM/SAH is reduced and inhibits DNMTs activity that depends on the intracellular availability of SAM as methyl donor [112]. Navarro-Perak et al. [113] suggested that EGCG inhibits DHFR (dihydrofolate reductase) and/or folic acid uptake, acting as an antifolate compound. Apart from SAM, folates are an important source of the 1-carbon unit used to methylate DNA. Low folates in cancer cells induced by EGCG administration prevent DNA hypermethylation of specific genes. The effective concentration of EGCG on human DHFR inhibition is around 33 nM, a value physiologically achievable in human plasma after ingestion of standardized preparation of GTCs [114,115,116,117]. One of the most important mechanisms to control gene expression depends on the chromatin condensation status, which is regulated by different processes, with histone acetylation/deacetylation being one of the most studied and finely regulated [105]. Histone acetylation often correlates with chromatin relaxation. Inhibition of histone deacetylases (HDACs) in cancer cells causes the re-expression of genes epigenetically silenced during carcinogenesis [118]. Polyphenon E^®^ administration has been reported to cause inhibition of HDACs activity and expression in PCa cell lines [106,119]. Molecular modeling and docking studies in HeLa cells suggested that EGCG inhibits HDAC1 activity by direct binding of the enzyme [108].

miRNAs are functional small non-coding RNAs that control gene expression by inducing degradation or translational inhibition of their target mRNAs. It has been demonstrated that various miRNAs are often overexpressed in cancer cells, including PCa cells, and their altered expression is associated with cancer progression [120]. miRNA-21 is regulated by androgens and promotes disease progression toward a hormone-independent phenotype [121]. Siddiqui et al. demonstrated that EGCG treatment of nude mice subcutaneously implanted with 22Rv1 PCa cells inhibited tumor growth by down-regulating miRNA-21 and induced the up-regulation of miRNA-330, a tumor suppressor that induces apoptosis in PCa cells [122].

### 3.3. Effects of GTCs on Protein Homeostasis

Tumor cells are continuously exposed to stressful conditions (such as hypoxia, nutrient deprivation, acidosis, chemotherapeutic treatments, etc.) and are characterized by a high translational activity; therefore, they are much more prone to accumulate misfolded and/or unfolded proteins. This stress is even higher in PCa secretory epithelial cells, which are characterized by a high rate of protein synthesis. For the maintenance of protein homeostasis (proteostasis), eukaryotic cells have developed a complex protein quality control system, which comprises molecular chaperons, the ER-associated degradation (ERAD) mechanism, the unfolded protein response (UPR), and the two most important systems of protein degradation, i.e. the proteasome system and the autophagic mechanism. Proteostasis impairment and accumulation of misfolded proteins in the ER lumen induce ER stress, which in turn activates the UPR, a complex network of molecular signaling to adapt and respond to ER stress conditions, thereby promoting cell survival. However, under chronic stress when proteostasis cannot be restored, the UPR activates apoptosis signaling that commits cells to death [123,124].

GTCs have been proven to trigger anticancer effects by targeting HSPs and proteasome functions and by interfering with the autophagic flux.

#### 3.3.1. Heat Shock Proteins

Heat shock proteins (HSPs) are a superfamily of molecular chaperones representing the first line of defense against protein misfolding and aggregation. They aid in the folding and refolding of proteins, and target denatured proteins to degradative systems. HSPs function both in physiological and pathological conditions. Different cellular pathways, associated with cancer onset and progression, may be involved in the activation of the heat shock factor-1 (HSF-1), the primary factor associated with HSPs gene activation [125,126]. The expression of different members of HSPs is modulated during PCa progression [127]. It is documented that EGCG inhibits carcinogenesis thanks to the down-regulation of both HSP90 and HSP70 and through the dissociation of their complexes with co-chaperones or client proteins [128]. In different cell lines that mimic progressive stages of PCa, Moses and colleagues demonstrated that EGCG inhibited cell proliferation and induced apoptosis by direct binding to HSP90, causing instability and altering the functions of HSP90-client proteins involved in tumor progression [129].

#### 3.3.2. Proteasome Inhibition

The ubiquitin-mediated proteasomal degradation is essential for the regulation of several cellular processes. The proteasome is a multicatalytic protein complex present in all eukaryotic cells, and it is responsible for the degradation of the major part of the cellular proteins. The chymotrypsin-like activity of the proteasome is associated with the tumor cell survival [130] since most of the intracellular proteins involved in the onset and progression of tumor are degraded through the ubiquitin-proteasome pathway. GTCs are strong and selective inhibitors of the proteasomal chymotrypsin-like activity in vitro (IC_50_ = 86 ÷ 194 nM) and in intact PCa cells (1 ÷ 10 µM). EGCG, among all the catechins tested, showed the strongest inhibitory activity [130]. The ester bond contained in galloylated catechins is required for potent inhibition of the proteasomal activity [130,131]. Other structure-activity relationship studies evidenced that the A ring and the gallate ester/amide bond are involved in the mechanism of proteasome inhibition [132]. Similar effects have been described for two synthetic enantiomeric analogs of natural GTCs in PCa cells [133]. The observed accumulation of short-lived proteins, normally addressed to proteosomal degradation, such as p21, p27, Bax, and ikBα in PCa cells after EGCG or GTCs treatment indirectly confirms that GTCs exert an inhibitory effect on the proteasome activity [98,134].

#### 3.3.3. Autophagy

The catabolic process through which long-lived proteins, damaged organelles, and other unnecessary intracellular materials are delivered to lysosomes for degradation is called autophagy. Normally autophagy is constitutively active at low levels to guarantee cellular homeostasis, but it can be strongly induced in stressful conditions when this process seems to act primarily as a cellular protective response [135]. The effects of EGCG on autophagy seem to be dependent on the concentration used, cell type, and stress conditions.

We have observed at a morphological level, by electron microscopy, that GTCs administration (0.3% in tap water) in TRAMP mice drastically reduced the condensing and secretory activities of the ER of prostate cells, causing an imbalance of the protein trafficking and an engulfment of ER, finally leading to ER stress. No effects were observed in age matched animals that were water fed. In agreement with morphological data, we found that the biogenesis of secreted proteins is altered in TRAMP-C2 cells treated with 20 µg/mL of GTCs [136]. We reported that autophagy was transiently activated in human prostate PNT1a cells, commonly used to mimic the initial stages of PCa, as a survival response to overcome Polyphenon E^®^-induced ER stress [42]. Treatment of PNT1a with 35 µg/mL of Polyphenon E^®^ for 24 h committed cells to apoptosis, while 145 µg/mL Polyphenon E^®^ committed metastatic prostate cancer PC3 cells to caspase-independent programmed cell death [42].

## 4. GTCs in Human PCa Chemoprevention: From Epidemiological Data to Interventional Studies

Three large cohort prospective studies, all conducted in the Japanese population, aimed to examine the association between dietary intake and PCa risk [137,138,139]. All these observational studies did not find any statistically significant correlation between green tea consumption and PCa risk. A fourth study examined the association between green tea consumption and risk of PCa stratified by disease stage [140] and corrected the results for potential confounder factors. The authors did not find a significant association between green tea consumption and the global risk of being diagnosed with PCa. Nonetheless, a dose-dependent inverse relation for the risk to develop advanced PCa for men who consumed five cups of green tea/day compared with those who consumed one cup/day was observed. Two case-control studies examined the relation between green tea intake and PCa in patients diagnosed with PCa (histologically confirmed) and PCa free matched hospital inpatients as controls [141,142]. Both these studies found an inverse relation between green tea consumption and PCa risk. Nonetheless these results should be interpreted very carefully due to many critical points that include the small sample size, the hospital based case-control design, the simultaneous consumption of green and black teas, differences in tea brewing methods, and possible interferences from other dietary factors. Although epidemiological evidences are still not conclusive, the anticancer efficacy of green tea is fully displayed in the TRAMP model when intervention is started early, at weaning, and the malignancy is not fully developed. In this chemopreventive scenario, concentrations of standardized green tea extract ranging between 0.1 ÷ 0.3% in drinking water are effective to strongly delay or stop PCa growth [12,15,92]. Adhami at al. demonstrated that green tea extracts are more effective when feeding (0.1% in drinking water) is started at very early stages of PCa development (presence of “in situ” histological lesions), while the chemopreventive potential decreases in animals bearing tumors of a more advanced stage, finally resulting in largely ineffective in animals that display moderately differentiated adenocarcinoma and occasionally metastasis [143]. To reinforce the concept that GTCs are ineffective as chemotherapeutic molecules, very limited or no effects have been observed in patients with advanced PCa [144,145,146]. At difference, we introduced for the first “Proof of Concept” that secondary chemoprevention of PCa with GTCs is feasible [17]. We enrolled 60 Italian patients (all having a Caucasian genetic background) diagnosed with HG-PIN, a premalignant lesion associated with a 30% increased risk to develop PCa within 1 year [147]. 30 volunteers were randomly assigned to receive, according to a double-blind procedure, 600 mg/day of a standardized GTCs formulation (total catechins 75.7%; EGC, 5.5%; EC, 12.2%; EGCG, 51.9%; ECG, 6.12%), divided in three capsules of 200 mg each, or identical placebo capsules for one year. All the patients, during the study, were subjected to regular prostate biopsy (6 and 12 months after the study began) to assess the differences in PCa rates between the two arms. After one year of treatment, only one tumor was diagnosed among the 30 GTCs-treated men, while nine cancers were found among the 30 placebo-treated men. No significant side or adverse effects were documented throughout the whole study. A randomized, placebo-controlled trial evaluated whether 400 mg of Polyphenon E^®^ significantly reduce PCa rates in a cohort of men diagnosed with HG-PIN and/or atypical small acinar proliferation (ASAP) [148]. 74 men (36 GTCs and 38 placebo) completed the one year protocol. No significant reduction of PCa incidence was observed between the two arms. Possible explanations of the discrepancies between the results recorded by Kumar et al. and those previously published by us may reflect differences in the study design, which include enrolment criteria (more heterogeneous) and GTCs posology (a lower dosage). Pathologists usually formulate ASAP diagnosis when it is not possible to find alterations in the cells’ morphology to support “without doubts” a PCa diagnosis. For this reason, ASAP is often considered an under-diagnosed cancer [149]. Men bearing both ASAP and multifocal HG-PIN are very likely patients in whom, at the moment of the first biopsy, PCa has already developed, even if not clearly supported by histopathological diagnosis [150]. We might hypothesize that in the study by Kumar et al. were included men for which the narrow window of time for therapeutic intervention with GTCs was already expired at the enrolment. As a consequence of the small number of patients enrolled, unfortunately, the study did not have sufficient statistical power to detect a reduction of PCa incidence in the subgroup of men diagnosed only with HG-PIN receiving GTCs in comparison with HG-PIN patients receiving the placebo group [148].

## 5. GTCs Bioavailability and Metabolism in Humans

Flavanols bioavailability in vivo is strongly limited by their high molecular weight and by a bulky hydration shell that magnifies the effective molecular size. Moreover, extensive enteroluminal biotransformation and phase II metabolism of flavan-3-ols, which includes methylation, glucuronidation, and O-methylation may affect GTCs bioactivity in vivo and explain inconsistent results in comparison to effects observed in vitro, using supraphysiological doses of EGCG [151]. Chow et al. investigated the pharmacokinetic of GTCs following single administration of Polyphenon E^®^ at increasing concentrations ranging from 200 mg to 800 mg or equivalent doses of pure EGCG [116,152]. The average EGCG peak plasma concentration (Cmax) was reached after 2–4 h from ingestion and declined rapidly, with very low/undetectable levels at 24 h after dosing. EGCG peak concentration increased with increasing doses in a nonlinear fashion, reaching a value above 1 µM at the highest concentration tested, possibly due to the existence of saturable mechanisms that cause a presystemic elimination of orally administered GTCs. EGC and EC were detected in plasma and urine samples after Polyphenon E^®^ administration, predominantly as glucuronic acid/sulfate conjugates, while EGCG was detected prevalently in its unchanged form. The presence of other catechins in the formulation tested does not influence EGCG plasma concentration, at difference the presence of food in the stomach interferes with catechins absorption and reduces threefold the plasmatic concentration of free catechins (EGCG, EGC, EC) [116,152]. The same authors observed that the systemic availability of free EGCG increases by 60% after repeated administration (four weeks of treatment) of a high daily bolus dose (800 mg once daily) and that this concentration is safe and well tolerated in healthy human subjects [116]. Similar results of catechins serum levels were obtained in patients diagnosed with PCa and receiving 800 mg of Polyphenon E^®^ for four to six weeks before scheduled prostatectomy surgery [146]. The authors instead found a very low to undetectable level of catechins in the prostate surgical samples collected from the same patients, possibly due to a combination of rapid systemic clearance (less than 24 h) and low bioaccumulation of polyphenols in the tissue [146]. Different GTCs concentrations ranging from 21 pmol/g to 107 pmol/g tissue were found in the prostate of men who consumed 1.42 L of green tea for five days before prostatectomy [153]. In human prostate glands of PCa patients who consumed six cups of green tea daily for up to six weeks before prostatectomy, EGCG is detectable mainly in the free form (nonglucuronidated/sulfated) [154]. Approximately half of the EGCG was present in the methylated form as 4″-MeEGCG. Results obtained in LNCaP cells indicated that methylation of EGCG may occur directly in prostate tissue catalized by cytosolic COMT. None of the nongallated GTCs, including EGC and EC or their metabolites, were found in the prostate tissue, indicating a lower bioavailability for these compounds in comparison with EGCG [154].

Catechins that are not absorbed in the small intestine, as well as conjugated catechins excreted in the bile, reach the large intestine, where they may be metabolized by colonic bacteria [151]. (−)-EC, (−)-EGC, and (−)-EGCG, are degradated by the human colon microbiota to 5-(3’,4’,5’-trihydroxyphenyl)-γ-valerolactone, 5-(3’,4’-dihydroxyphenyl)-γ-valerolactone, and 5-(3’,5’-dihydroxyphenyl)-γ-valerolactone. Some of these colonic metabolites may enter the circulation and undergo further modifications before being excreted with urine in quantities corresponding to about 40% of total ingested GTCs [155]. Colonic biotransformation represents, therefore, a significant component of the overall bioavailability equation and significantly influences GTCs bioactivity. To the best of our knowledge, no data are available on the bioactivity of these compounds on PCa cells.

## 6. Nanotechnology Strategies to Improve GTCs Bioactivity

The great potential of use of GTCs or EGCG as safe natural molecules to prevent PCa onset or eventually revert early PCa lesions is limited by the intrinsic chemical instability, low bioavailability, and quick in vivo clearance of these products. Drug formulation strategies aimed at improving GTCs/EGCG stability assist efficient and specific delivery at target cells and limit undesired metabolism and are therefore expected to produce a valuable benefit in terms of an improved pharmacodynamic profile. The concept of nanochemoprevention, i.e. the uses of nanotechnology for enhancing the outcome of chemoprevention, was first introduced by Siddiqui et al. [156]. NPs are nanocarriers made of biodegradable/biocompatible materials sized between 1 nm and 100 nm, used as vehicles to improve drug delivery. Different types of NPs have been used to improve the pharmacological profile of EGCG, including gold nanoparticles, polymeric nanoparticles, liposomes, carbohydrates, and inorganic nanoparticles [157,158].

Siddiqui et al., in their “Proof of Principle”, study demonstrated that EGCG encapsulated in polylactic acid (PLA)–polyethylene glycol (PEG) nanoparticles has an improved efficacy against human PCa cells both in vitro and in vivo [156]. The inhibition of PC3 proliferation by half of normal values, measured as the IC_50_, was found to be tenfold lower when nano-EGCG was used in comparison to non encapsulated EGCG. In vivo, a tenfold lower dose of nano-EGCG was required to achieve a similar extent of xenograft tumors to that obtained with EGCG.

Polymeric EGCG-encapsulated NPs, functionalised with a mimetic ligand of the prostate specific membrane antigen (PSMA), shows an improved selective EGCG delivery in PSMA expressing PCa cells, thus leading to a more marked inhibition of cell proliferation [159,160]. The same authors observed a significant increase of tumor growth inhibition in mouse xenografts treated with PSMA targeted NPs, in comparison to EGCG. This effect may be attributed to the selective binding of NPs to PSMA, which presumably would promote an active targeting through receptor-mediated endocytosis [160]. One disadvantage of using PLA-PEG NPs is their low stability in the acidic environment that precludes the oral route of administration. Chitosan NPs encapsulating EGCG (Chit-nanoEGCG) at difference are characterized by the slow release of EGCG in simulated gastric juice acidic pH and a faster release in simulated intestinal fluid. Treatment with Chit-nanoEGCG resulted in significant growth inhibition of subcutaneously implanted 22Rν1 PCa xenografts. In tumor tissues of mice treated with Chit-nanoEGCG, compared with animals treated with EGCG, there was a significant induction of apoptosis and reduction of cell proliferation [161].

EGCG was efficiently loaded in polysaccharides NPs made of Arabic gum and maltodextrin. Encapsulated EGCG retained its biological activity; indeed it reduced cell viability and induced apoptosis when administered to DU145 PCa cells. Colony formation assay showed that nanoencapsulated EGCG possesses an enhanced inhibitory effect on cell proliferation at physiologically achievable concentrations (1 ÷ 2 µM), compared with free EGCG [162].

EGCG encapsulated in solid lipid nanoparticles (EGCG-SLN) increases molecule stability in aqueous solution, improves efficient cell delivery, and yields a fourfold increase in cytotoxicity against DU145 human PCa, in comparison to cells that received pure EGCG at the same concentration [163].

EGCG intercalated into Ca/Al-NO_3_ layered double hydroxide (LDH) NPs produced a higher inhibition of proliferation and colony formation suppression in PC3 cells, compared to EGCG alone. Similarly, enhanced apoptosis was observed in PC3 treated with EGCG-LDH in comparison to non-encapsulated EGCG. The enhanced antitumor activity of EGCG-loaded LDH-NPs is well correlated to the mechanism of diffusion of the EGCG through the vehicle that allowed a sustained gradual release of the drug in solution [164].

EGCG can also be used as a “Trojan horse” to deliver anticancer therapy specifically in PCa cells. Recently, it has been demonstrated that EGCG functionalized radioactive biocompatible gold NPs, derived from the Au-198 isotope (EGCG-198AuNPs), selectively bind with high affinity to the 67-LR, a receptor that is overexpressed in PCa cells. Significant amounts of EGCG-198AuNPs were internalized through 67-LR receptor endocytosis in tumor cells. Pharmacokinetic studies in PC-3 xenograft SCID mice showed a high retention of EGCG-198AuNPs in tumors and an 80% reduction of tumor volumes, compared to controls [80]. EGCG and gelatin-doxorubicin conjugate (GLT-DOX)-coated gold NPs (GLT-DOX/EGCG AuNPs) were produced to improve the specific uptake of doxorubicin in PC3 cells using EGCG/67-LR receptor-mediated delivery. Apoptosis of GLT-DOX/EGCG AuNPs treated PC3 was improved in comparison with cells treated with DOX [165].

## 7. Conclusions

Many authors have suggested that the anticancer effects of GTCs might be achieved through their direct ability to scavenge harmful ROS/RNS, or alternatively, through their autoxidation, which, in turn, produces H_2_O_2_, a toxic compound. Both these effects, observed in vitro, have been obtained using a supraphysiological concentration of GTCs and are very unlikely to take place in vivo. Recent achievements in this field of research have clarified that GTCs can interact with molecular targets that are specifically deregulated in PCa, such as the 67-LR receptor, at nanomolar concentrations. Others have demonstrated that GTCs modify the expressions of genes relevant for PCa progression, acting on transcription factors and through epigenetic mechanisms, or activating complex adaptive signaling such as the UPR. Despite the fact that GTCs have been proven to effectively inhibit PCa progression in animal models, their chemopreventive action in human PCa is still debated. The lessons learnt from clinical trials so far indicate that the therapeutic window for GTCs intervention is very narrow and possibly corresponds to early signs of prostate tissue transformation such as HG-PIN. Moreover inadequate dosage and/or short intervention periods may compromise the therapeutic effect. It is now clear that individual differences in GTCs metabolism and absorption may negatively affect the already low bioavailability, consequently reducing the bioactivity. New opportunities to improve the therapeutic value of GTCs come from the field of the nanotechnologies, which, when applied to chemoprevention, lead to the development of nanochemoprevention strategies. More than encouraging results have shown that biopolymeric NPs help to selectively deliver GTCs in PCa cancer cells, improving drug stability and increasing in vivo bioactivity. Large, well-designed, interventional studies are urgently needed to evaluate the efficacy of GTCs charged NPs in PCa prevention.
